# Spores of *Clostridium* engineered for clinical efficacy and safety cause regression and cure of tumors *in vivo*

**DOI:** 10.18632/oncotarget.1761

**Published:** 2014-01-12

**Authors:** John T. Heap, Jan Theys, Muhammad Ehsaan, Aleksandra M Kubiak, Ludwig Dubois, Kim Paesmans, Lieve Van Mellaert, Richard Knox, Sarah A. Kuehne, Phillipe Lambin, Nigel P. Minton

**Affiliations:** ^1^ Clostridia Research Group, Centre for Biomolecular Sciences, School of Life Sciences, The University of Nottingham, University Park, Nottingham, UK; ^2^ Maastro Lab, Research Institute GROW, University of Maastricht, MD Maastricht, The Netherlands; ^3^ Molecular Bacteriology, Rega Institute for Medical Research, University Leuven, Minderbroedersstraat,Belgium; ^4^ Morvus Technology Limited, Ty Myddfai, Llanarthne, Carmarthen, UK; ^5^ Present address: Centre for Synthetic Biology and Innovation, Department of Life Sciences, Imperial College London, London, UK

**Keywords:** Clostridia, spores, hypoxia, pro-drug converting enzyme, nitroreductase, CB1954, solid tumor

## Abstract

Spores of some species of the strictly anaerobic bacteria *Clostridium* naturally target and partially lyse the hypoxic cores of tumors, which tend to be refractory to conventional therapies. The anti-tumor effect can be augmented by engineering strains to convert a non-toxic prodrug into a cytotoxic drug specifically at the tumor site by expressing a prodrug-converting enzyme (PCE). Safe doses of the favored prodrug CB1954 lead to peak concentrations of 6.3 μM in patient sera, but at these concentration(s) known nitroreductase (NTR) PCEs for this prodrug show low activity. Furthermore, efficacious and safe *Clostridium* strains that stably express a PCE have not been reported. Here we identify a novel nitroreductase from *Neisseria meningitidis*, NmeNTR, which is able to activate CB1954 at clinically-achievable serum concentrations. An NmeNTR expression cassette, which does not contain an antibiotic resistance marker, was stably localized to the chromosome of *Clostridium sporogenes* using a new integration method, and the strain was disabled for safety and containment by making it a uracil auxotroph. The efficacy of *Clostridium*-Directed Enzyme Prodrug Therapy (CDEPT) using this system was demonstrated in a mouse xenograft model of human colon carcinoma. Substantial tumor suppression was achieved, and several animals were cured. These encouraging data suggest that the novel enzyme and strain engineering approach represent a promising platform for the clinical development of CDEPT.

## INTRODUCTION

Biotechnology potentially offers unconventional routes to new cancer therapies, and research in this area has focused on using viruses as vectors to deliver therapeutic genes to tumors. Unfortunately, issues with the safety of viral vectors have been encountered [1, 2] and only recently has a virus system showing good specificity and tumor infiltration been described [3]. Several types of bacteria have also been investigated as delivery vectors, and as therapeutics in their own right [4, 5]. Spores of some species of *Clostridium*, which are strictly anaerobic bacteria, naturally target tumors with high specificity following intravenous administration, because the dormant spores can germinate and grow only in the hypoxic/necrotic cores of solid tumors [6-8] which are difficult to target using viral vectors [9, 10]. The growing bacteria secrete proteases inside the tumor, rapidly digesting the tumor mass. This approach is especially interesting because it directly targets the hypoxic cells in poorly vascular regions which are refractory to conventional treatments. Any *Clostridium* cells entering normal tissue from a colonized tumor would be poisoned by oxygen and die.

**Figure 1:**
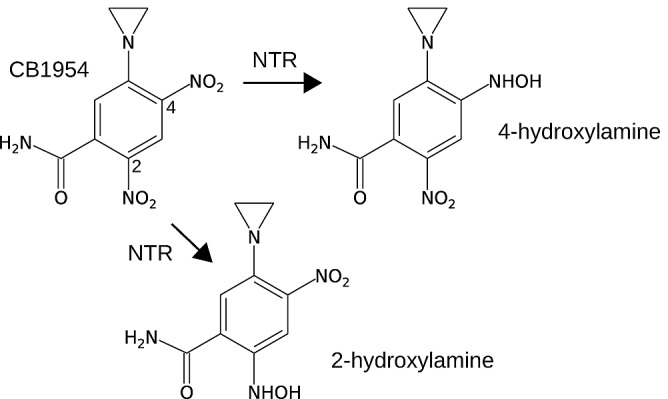
The prodrug CB1954 is a low-toxicity monofunctional DNA-alkylating agent, but can be converted to a much more toxic bifunctional DNA-alkylating agent upon 2 x 2-electron reduction of the 4-nitro or 2-nitro group to the corresponding hydroxylamine The 4-hydroxylamine is more cytotoxic than the 2-hydroxylamine. Some NAD(P)H-dependent nitroreductase (NTR) enzymes can catalyse this reaction, with varying kinetics and nitro group specificity (see Table 1).

Stringent spacial containment by oxygen is an excellent safety feature, absent from treatments using bacteria that are not strict anaerobes [11, 12], but it also allows neoplastic cells at the oxygenated tumor periphery to escape proteolysis [13]. Therefore, we and others have sought to augment the anti-tumor effect of *Clostridium* strains by genetically modifying them to express therapeutic proteins, mainly enzymes which sensitize the tumor to specific chemotherapeutic agents [14-20]. The prodrug CB1954 (5-(aziridin-1-yl)-2,4-dinitrobenzamide) shown in Figure 1 is an attractive candidate because its clinical safety has been demonstrated [21], and because a 10,000-fold increase in toxicity is achieved upon its activation by a suitable nitroreductase (NTR) enzyme to the 4-hydroxylamine (4HX) derivative 5-(aziridin-1-yl)-4-hydroxylamino-2-nitrobenzamide. The 4HX derivative forms a potent DNA cross-linking agent [22, 23] which, importantly, has been shown to kill both proliferating and non-proliferating cells [24], both of which are present in hypoxic tumor areas.

**Table 1:** CB1954 nitroreductase properties of selected enzymes

Enzyme	K_cat_ (s^−1^)	K_m_^CB1954^ (μM)	K_cat_ /K_m_^CB1954^ ( s^−1^ μM^−1^)	K_m_^NADH^ (μM)	Products	Source / Comment
NmeNTR	4.04 (NADH) 15.23 (NADPH)	2.47	1.636 (NADH) 6.166 (NADPH)	188	4HX	NTR from *Neisseria meningitidis* MC58 (this study). Lowest K_m_^CB1954^ and highest K_cat_ /K_m_^CB1954^ values of any reported enzyme.
HsoNTR	3.5 (NADH) 13.13 (NADPH)	22.03	0.159 (NADH) 0.596 (NADPH)	644	4HX	NTR from *Haemophilus somnus* 129PT (this study).
NfnB	6	862	0.007	6	4HX + 2HX	Widely-studied *E. coli* NTR (Knox et al., 1992).
NfnB N71S/F124K	7.5	170	0.044	5	4HX + 2HX	Mutant obtained by protein engineering (Searle et al., 2004 and Race et al., 2007).
HinNTR	56.2	690	0.081	ND	4HX	NTR from *Haemophilus influenzae* (Theys et al., 2006). Highest K_cat_ value of any reported enzyme.
NfsA	2.60 (NADH) 20.9 (NADPH)	18 (NADH) 140 (NADPH)	0.15	ND	2HX	A second *E. coli* NTR (Vass et al., 2009).
BliNTR	6.4	30	0.213	820	4HX	NTR from *Bacillus licheniformis* (Emptage et al., 2009.)
FRase I F124W	26.6	259	0.103	ND	4HX	Mutant of FRase I from *Vibrio fischeri* obtained by protein engineering (Swe et al., 2012).

The enzymes shown include those with the most favourable values reported for K_cat_, K_m_^CB1954^, and K_cat_ /K_m_^CB1954^.

Encouraging preclinical efficacy data have been achieved with genetically engineered NTR-expressing *Clostridium* [19, 20]. However, for this *Clostridium*-Directed Enzyme-Prodrug Therapy (CDEPT) using CB1954 to be suitable for clinical evaluation, two issues must be addressed. Firstly, the poor kinetics of CB1954 activation by known NTRs must be improved upon. The maximum concentration of CB1954 which can be safely achieved in patient serum is only 6.3 μM [21], at which concentration previously-reported enzymes are not saturated. Secondly, a recombinant *Clostridium* strain must be constructed with properties appropriate for clinical application. The DNA encoding the prodrug-converting enzyme should be stably localized to the chromosome rather than carried by a plasmid, antibiotic-resistance gene(s) used during strain construction must be removed, and preferably the organism should be disabled to prevent its growth in the event of a release into the environment. A strain meeting these criteria has not been described in any report to-date. Here we solve each of these problems and demonstrate the efficacy of CDEPT in a mouse xenograft model of human colon carcinoma, using CB1954 and a recombinant *Clostridium* strain potentially suitable for clinical trial.

## RESULTS

### A novel NTR which efficiently activates CB1954 at clinically-achievable concentrations

Known enzymes with NTR activity against CB1954 are NAD(P)H-dependent quinone reductases belonging to one of two large protein families, Pfam PF00881 ‘Nitroreductase’ and Pfam PF02525 ‘Flavodoxin_2’, such as *E. coli* NfnB [25] and *Bacillus amyloliquefaciens* YwrO [26] respectively. We identified homologs of these proteins in the genome sequences of organisms or genomic DNA available in our laboratory (see Supporting Information), and investigated the activity of several of these against CB1954. Each gene encoding a candidate CB1954-activating enzyme was cloned in an *E. coli* expression vector. Crude cell lysates were used in initial screens for functional expression, and then to test for CB1954 nitroreductase activity using an HPLC assay (see Supporting Information). CB1954 was rapidly consumed by cell lysates containing the NfnB homolog from *Haemophilus somnus* 129PT or *Neisseria meningitidis* MC58. Therefore these two enzymes, which we designate HsoNTR and NmeNTR respectively, were studied in more detail.

NmeNTR and HsoNTR were each affinity-tagged, over-expressed, purified, and the CB1954 nitroreductase activities of the purified proteins characterized using the HPLC assay. The reaction products and kinetic parameters of these enzymes with respect to CB1954 and NADH were determined (Table 1). Both NmeNTR and HsoNTR reduce CB1954 only at the 4-nitro group, producing the highly toxic 4HX derivative. This contrasts with the widely-studied *E. coli* NfnB which reduces CB1954 at either the 4-nitro group or 2-nitro group, producing equimolar quantities of the 4HX derivative and much less toxic 2-hydroxylamine (2HX) derivative (5-(aziridin-1-yl)-2-hydroxylamino-4-nitrobenzamide) [27, 28]. Moreover, the kinetic parameters of HsoNTR and NmeNTR differ dramatically from those of *E. coli* NfnB and other published enzymes (Table 1). Crucially, NmeNTR is the first reported enzyme with a *K_m_* ^CB1954^ lower than 6.3 μM, the maximum concentration of CB1954 which can be safely achieved in patient serum. NmeNTR would therefore approach saturation with CB1954 in a therapeutic regime using the maximum clinically-recommended dose, so the prodrug would be efficiently activated and high levels of tumor-specific cytotoxicity achieved.

### Construction of NTR-expressing *Clostridium* strains that are stable, disabled, and lack antibiotic marker genes

To establish whether the apparently superior kinetic properties of NmeNTR would result in improved therapeutic performance, we sought to construct and compare strains of *Clostridium sporogenes* NCIMB 10696 expressing either NmeNTR or the most-studied CB1954-activating enzyme *E. coli* NfnB in an *in vivo* model. *C. sporogenes* NCIMB 10696 is one of the tumor-targeting *Clostridium* strains currently most studied as a potential therapeutic agent [16-20] along with *Clostridium novyi* NT [29-32].

In previous CDEPT studies nitroreductases have been expressed using autonomous plasmids, but these exhibit segregational instability [33], causing the proportion of plasmid-containing cells in a population to decrease during growth. Therefore expression of a plasmid-borne NTR is likely to be poor and heterogenous in a tumor colonized through many generations of bacterial growth [34] following the initial infiltration of a small number of plasmid-containing spores. Plasmids also carry a risk of transfer to other organisms. To overcome these issues, we opted to use expression constructs localized to the chromosome, which has complete segregational stability and no mechanism for transfer. The insertion of heterologous expression constructs into the chromosome of tumor-targeting *Clostridium* sp. has not previously been reported, but is now possible using methods for chromosomal integration in *C. sporogenes* that we have recently developed [35-37].

An expression cassette was constructed using a strong, constitutive promoter derived from the ferredoxin gene *fdx* of *C. sporogenes* to direct expression of NmeNTR from a synthetic coding sequence sequence with codon usage optimized to suit *C. sporogenes*. A similar expression cassette for *E. coli* NfnB was also constructed. The NmeNTR expression cassette was initially inserted into the *C. sporogenes* chromosome using the ClosTron method [35, 36] which employs a modified bacterial Group II intron (a type of mobile retro-element [38]) to deliver the sequence of interest. Recombinant clones were isolated using the antibiotic erythromycin, resistance to which is conferred by the marker gene *ermB* within the insertion. As antibiotic resistance is undesirable in therapeutic strains, subsequent removal of *ermB* from the chromosome using FLP recombinase was attempted, but was unsuccessful (see Supporting Information).

**Figure 2:**
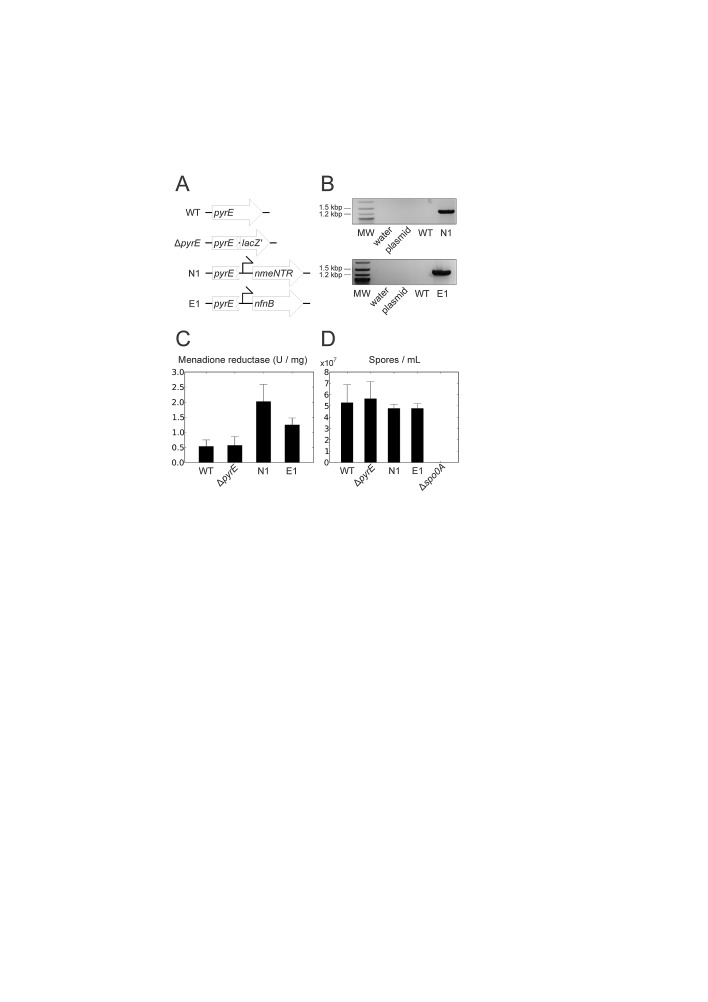
Construction and *in vitro* characterization of *C. sporogenes* therapeutic strains (*A*) Chromosomal *pyrE* locus of therapeutic strains and control strains. WT, wild type; Δ*pyrE*, a mutant constructed previously by integrating the ‘empty’ cloning site (*lacZ*') of pMTL-JH27 [37]; N1, the strain containing a NmeNTR expression cassette; E1, the strain containing an *E. coli* NfnB expression cassette; angled arrows, *fdx* promoter. (*B*) Successful integration shown by PCR using chromosome-specific primer Csp-pyrD-sF2 in combination with insert-specific primer M13F. MW, 2-Log DNA Ladder (NEB) molecular weight marker; plasmid, pMTL-JH27 derivative containing NmeNTR or NfnB cassette accordingly; WT, wild-type *C. sporogenes* genomic DNA; N1 and E1, genomic DNA from strain N1 or E1 respectively. (*C*) Functional expression of NmeNTR and NfnB, in strains N1 and E1 respectively, indicated by specific menadione reductase activities in cell lysates. Data shown are the mean of three independent experiments, error bars show standard deviation. N1 and E1 show specific menadione reductase activities elevated above the background activity of endogenous quinone reductases seen in WT and Δ*pyrE*. (*D*) NTR-expressing and control strains are equally able to form spores. Strains were grown in complex medium (TYG) for five days, heated to inactivate vegetative cells, and spore titres were determined by plating. A non-sporulating Δ*spo0A* mutant constructed previously [35] is included as a control. Data shown are the mean of three independent experiments, error bars show standard deviation.

To circumvent the need for marker removal, we used our recently-developed integration method ACE, in which an antibiotic selection marker need not be incorporated into the chromosome along with the expression cassette [37]. NmeNTR and *E. coli* NfnB expression cassettes were inserted into the *C. sporogenes* chromosome using integration vector pMTL-JH27, which targets *pyrE*, a gene involved in pyrimidine biosynthesis. By inactivating *pyrE* during integration, recombinant cells are also uracil auxotrophs and effectively disabled from growth in the environment. The concept of disabling a strain to ensure containment was proposed at the advent of recombinant DNA technology [39] and has recently been applied to restrict *Salmonella* amino acid auxotrophs to tumors [11, 12]. Using this approach, the desired recombinant *C. sporogenes* strains containing *E. coli* NfnB and NmeNTR were readily obtained, and were designated E1 and N1, respectively (Figure 2*A* and *B*). The ‘empty’ vector pMTL-JH27 was previously used to generate a *pyrE* mutant without an expression cassette [37] which we used as a control in this study (Figure 2*A*).

### Strain characterization *in vitro*

To determine whether the heterologous nitroreductases were functionally expressed by strains E1 and N1, we measured the menadione reductase activity in cell lysates of these strains and compared them to the wild type and the *pyrE* mutant. Cells lysates of both E1 and N1 contained menadione reductase activity elevated significantly above the endogenous levels measured in the controls (Figure 2*C*).

The disablement of E1, N1 and the *pyrE* mutant was confirmed by sub-culturing cells of these strains and the wild type from the complex medium TYG onto plates of a defined growth medium either with or without supplementary uracil. The wild type grew equally well with or without the uracil supplement, whereas E1, N1 and the *pyrE* mutant were unable to grow without exogenous uracil, confirming their disablement, a uracil auxotrophic phenotype.

CDEPT depends upon the ability of *Clostridium* to form spores, the metabolically-dormant, highly-robust form suitable for intravenous administration and for long periods of storage. Therefore it was necessary to determine whether the ability of the recombinant strains to form spores had been affected by the inactivation of *pyrE*, the addition of the NTR expression cassettes, or the sub-culturing steps during the genetic modification procedure. The wild type, E1, N1 and the *pyrE* mutant were cultivated in TYG broth, in which the wild type forms spores over time. A *spo0A* mutant, which is completely unable to form spores, was also included as a control [35]. Samples taken over several days were heated to inactivate vegetative cells, and the titre of heat-resistant colony-forming units (corresponding to viable spores) was determined by dilution plating (Figure 2*D*). The *spo0A* mutant formed no colonies following heat treatment, confirming that the procedure inactivated all vegetative cells. E1, N1 and the *pyrE* mutant were all able to form spores, and did so at the same rate as the wild type, and to the same final titer.

### CDEPT treatment of tumors *in vivo*

**Figure 3:**
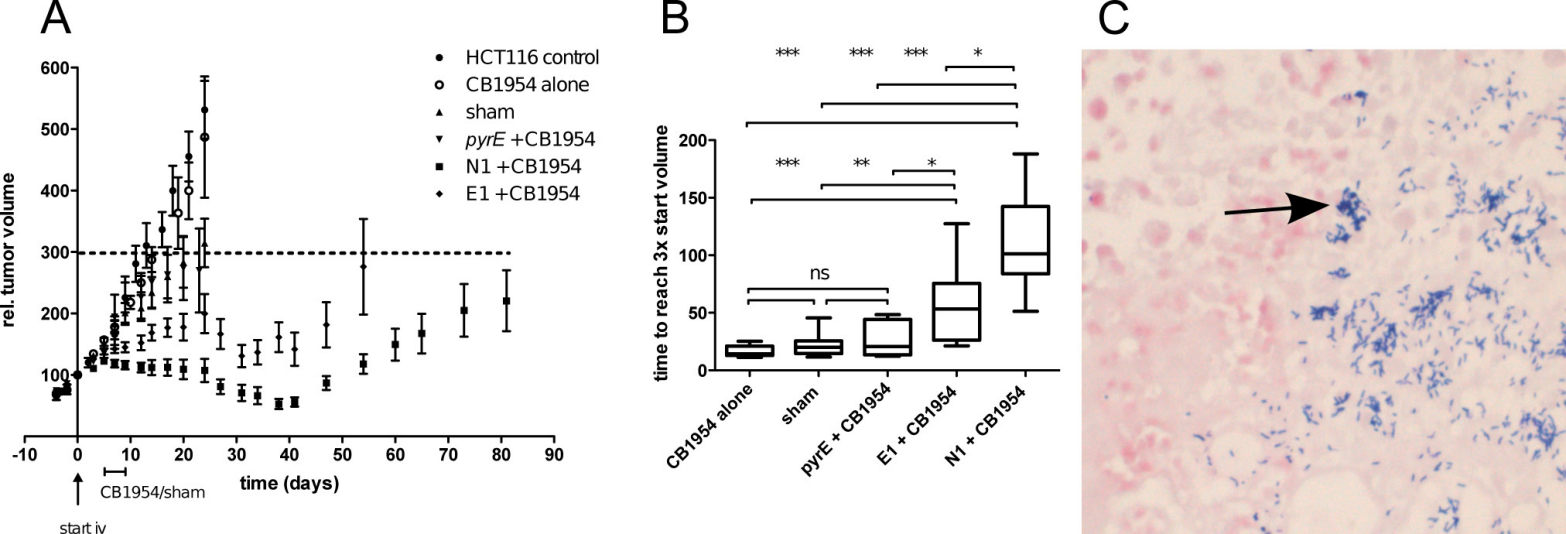
The NTR-expressing therapeutic strains are efficacious in CB1954 therapy *in vivo* (*A*) Growth curves of HCT116 tumors treated as indicated in the legend. Spores (5 x 10^7^) were injected on day 0 when tumors reached a volume of ~250 mm^3^ and CB1954 prodrug (15 mg/kg) or sham treatment was given for 5 consecutive days starting at day 5 post spore injection. Data shown are average ± SEM. (*B*) Growth delay of individual tumors in the different groups. Growth delay is defined as the time for the tumor to reach three times its volume at the start of the prodrug or sham treatment. Statistical differences between groups are calculated using the non-parametric Mann-Whitney test (*, p<0.05; **, p<0.01; *, *p<0.001) (C*) Representative gram staining of tumor sections showing colonization of the tumor by *C. sporogenes* vegetative cells following spore administration, germination in the tumor, and outgrowth. *C. sporogenes* vegetative cells are visible as gram-positive purple rods and reside in the necrotic area of the tumor. HCT116 nuclei (pink) are counterstained with iodine solution.

Since strains E1 and N1 express stably-integrated NTR under the regulatory control of a strong promoter without inclusion of any antibiotic resistance gene, we next determined whether administration of these clinically-applicable strains in combination with prodrug administration to tumor-bearing animals would result in anti-tumor efficacy. To do so, we compared the effect in animals that received (1) no treatment, (2) CB1954 prodrug alone, (3) sham treatment (spores and vehicle), (4) *pyrE* mutant control spores in combination with CB1954 and (5) N1 or E1 spores in combination with CB1954, each in a standard experimental tumor growth delay setting. This choice of groups allowed us to compare the contribution of the two major improvements reported here, stable integration and improved enzyme kinetics. Spores were collected, quantified and intravenously administered at a concentration of 5x10^7^ cfu per animal when tumors reached a volume of ~250 mm^3^, followed five days later by sham or prodrug administration. Colonization evaluation in randomly chosen tumors from each group (n≥14 per group) showed equal numbers of the *C. sporogenes* cells per g tumor tissues for all strains, with >99% of cfu being in the metabolically active vegetative form. Colonization was also confirmed by gram staining. In tumors, *C. sporogenes* vegetative cells are visible as purple, gram-positive rods (Figure 3*C*). Disablement of the strain by knocking out *pyrE* thus did not affect the excellent tumor-colonizing capacity of *C. sporogenes*. In line with our previous observations [19] administration of prodrug alone did not result in any effect, and only a small (non-significant) growth delay was observed upon administration of spores alone (sham) or control spores in combination with CB1954 (Figure 3 *A*). Strikingly, combining spores of strain E1 or N1 with CB1954 resulted in a significant growth delay, the effect being most pronounced for N1 (p<0.001, Figure 3 *B*). This suggests that the superior kinetic properties of NmeNTR, which allow conversion of the prodrug to its toxic derivative at a much lower concentration than *E. coli* NfnB, contribute substantially to the observed effect. The most striking effect we observed was the disappearance of several tumors in both CDEPT groups. In the E1/CB1954 group, two out of 16 tumors completely disappeared and in the N1/CB1954 four out of 16 tumors completely disappeared (Figure S1). During the treatment, a mild and transient weight loss could be observed and the animals recovered completely during further follow-up time.

## DISCUSSION

The poor activity of known nitroreductases at clinically-relevant CB1954 concentrations has led to several previous attempts to obtain an enzyme with more suitable kinetic properties, by screening native enzymes [26, 40-43] and/or protein engineering [43-47] with some success. However, NmeNTR, the *N. meningitidis* homolog of *E. coli* nitroreductase NfnB reported here, is the first example of an enzyme with a *K_m_*^CB1954^ value that can be exceeded by the clinically-achievable serum concentration of the prodrug. This *K_m_*^CB1954^ value (2.47 μM) is also seven-fold lower than the lowest previously reported (NfsA, 18 μM, Table 1). This key improvement makes NmeNTR much more suitable for clinical application than other known enzymes.

NmeNTR reduces only the 4-nitro group of CB1954, producing the highly-cytotoxic 4HX derivative, unlike *E. coli* NfnB which produces both the 4HX and 2HX derivatives in similar proportions [27, 28]. Among the enzymes whose CB1954 nitroreductase activity has now been characterized, specificity for the 4-nitro group appears to be typical (Table 1). *E. coli* NfnB, although the most widely studied CB1954 nitroreductase, is unusual in this respect. The 4HX derivative is far more cytotoxic than the 2HX derivative [22] which suggests that an enzyme such as NmeNTR with specificity for the 4-nitro group would lead to greater tumor-specific cytotoxicity.

By localizing expression cassettes to the chromosome using double crossover homologous recombination, we have constructed stable strains that lack both antibiotic resistance markers and any mechanism for transfer of the heterologous sequence. This feature represents a major improvement over previous studies in terms of efficacy and safety, as the instability of known plasmid replicons in *C. sporogenes* has been established [33] and transfer of natural and recombinant conjugal plasmids and transposons is known to occur in *C. sporogenes* and close relatives.

*C. sporogenes* is naturally confined to the hypoxic tumor core by oxygen, and the disabling *pyrE* mutation we introduce would serve to severely limit growth of modified strains in the environment in the event of accidental release from a clinical setting. Tumor containment and/or targeting features have been described for other recombinant bacterial strains with anti-tumor potential, such as *Salmonella* [11, 12]. The use of elegant synthetic regulatory mechanisms has also been proposed for containment [4] but we are cautious of their use for this purpose, and suggest that physiological mechanisms may be more appropriate. Any system in which containment depends upon the continued function of introduced component(s) will be vulnerable to failure through spontaneous loss-of-function mutations. Such mutations are inevitable at some frequency, and mutants would have a selective advantage for growth outside the tumor, so these systems would be inherently fragile. In contrast, no loss-of-function mutation is likely to allow an auxotrophic mutant of a strict anaerobe, as reported here, to escape the tumor environment.

Our approach to constructing stable, disabled, antibiotic marker-free strains of *Clostridium* expressing heterologous genes represents a platform for future efforts to build upon, as it provides a tumor-specific delivery system for the development of safe and effective gene therapies. The novel PCE NmeNTR in combination with CB1954 is a promising candidate. Besides cytotoxicity, strains could be engineered to have additional clinically-useful properties. For example, we are currently working to enable imaging of tumors undergoing *Clostridium* therapy via clinically-relevant imaging modalities such as PET. With the key strain engineering challenges overcome, a clinical trial to realize this therapy is now in prospect.

## METHODS

Full methods are given in SI Appendix, Methods.

### Enzyme characterization

Genes encoding homologs of *nfnB* and *ywrO* were PCR-amplified from genomic DNA or cell lysates. *E. coli* expression plasmids for each gene were constructed by standard methods. Menadione reductase activities of lysates of *E. coli* cells containing the expression plasmids were determined using a spectrophotometric assay, and CB1954 nitroreductase activities of lysates were determined using an HPLC assay. Specific activities were calculated using protein concentrations of lysates determined using the Bradford assay. The 4HX and 2HX derivatives of CB1954 were identified with reference to standards, their known absorbance spectra, and their anticipated column retention times. For determination of kinetic parameters using the same CB1954 nitroreductase assay, NTR expression plasmids were modified to encode C-terminal 6xHis tags, and enzymes were purified using a Novagen His-Bind kit.

### Construction of recombinant *C. sporogenes*

*C. sporogenes* was cultured anaerobically. Plasmids were transferred to *C. sporogenes* by conjugation from an *E. coli* donor as described previously [33]. Expression cassettes for NmeNTR and *E. coli* NfnB were constructed using derivatives of the *fdx* promoter from *C. sporogenes* and a synthetic, codon-optimized gene (Entelechon GmbH). Expression cassettes were initially inserted into the *C. sporogenes* chromosome using the *pyrF*-specific Group II intron plasmid pMTL007C-E2::Csp-pyrF-595s, and FLP-mediated removal of the *ermB* attempted as described previously [36]. Subsequently, expression cassettes were inserted into the *C. sporogenes* chromosome using the *pyrE* integration plasmid pMTL-JH27 as described previously [37].

### Characterization of recombinant *C. sporogenes*

Menadione reductase activities of lysates of *C. sporogenes* cells were determined to establish functional expression of the heterologous NTRs. Uracil auxotrophy / prototrophy was determined by sub-culturing fresh *C. sporogenes* colonies grown on the complex medium TYG onto plates of the defined growth medium MI either with or without supplementary uracil. Spores titres were determined as colony-forming units resistant to heat-treatment at 80 °C for 20 minutes.

### Mouse xenograft tumor model

Human colorectal HCT116 carcinoma cells were injected subcutaneously into adult NMRI nu/nu mice. Tumor volumes were determined three times per week using calipers. 5x10^7^ spores were administered when tumors reached a volume of ~250 mm^3^, and sham or prodrug treatment was started five days later. CB1954 was administered intraperitoneally at a concentration of 15 mg/kg for five consecutive days. At the end of the follow-up period, colonization levels were determined in tumors and normal tissues as described previously [48]. Tumor growth delay was determined as the time from the start of the treatment necessary for a tumor to triple in volume. Statistical significance was tested using a non-parametric Mann Whitney test. All animal testing was performed in accordance with local institutional guidelines and approved by the Animal Ethics Committee.

## SUPPLEMENTARY FIGURE AND TABLE






