# Supplementary material for: Spores of Clostridium engineered for clinical efficacy and safety cause regression and cure of tumors in vivo
Source: Oncotarget. 2014 Jan 12;5(7):1761–9. doi: 10.18632/oncotarget.1761 (PMC4039107; doi:10.18632/oncotarget.1761)
Supplement: Supplementary file 1 [file oncotarget-05-1761-s001.pdf]

Spores of *Clostridium* engineered for clinical efficacy and safety cause regression and cure of tumors *in vivo*- Heap et al

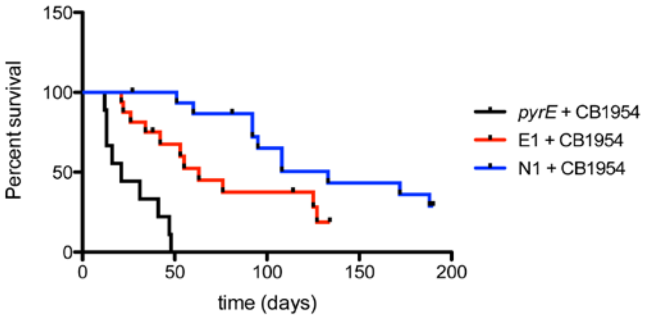

Figure S1**:** Kaplan-Meier curves showing time to reach the endpoint (three times the volume at start of i.v. spore injection) for mice injected with (a) *pyrE* control spores, (b) E1-expressing spores or (c) N1-expressing spores in combination with CB1954 prodrug administration. Statistical differences between groups are calculated using the Mantel-Cox test and equate to a difference of $p<0.001$ between *pyrE* vs E1, of $p<0.001$ between *pyrE* vs N1 and of $p=0.05$ for E1 vs N1. Animals in both N1 and E1 treatment groups not reaching the endpoint were all tumor-free.
