# Supplementary material for: Spores of Clostridium engineered for clinical efficacy and safety cause regression and cure of tumors in vivo
Source: Oncotarget. 2014 Jan 12;5(7):1761–9. doi: 10.18632/oncotarget.1761 (PMC4039107; doi:10.18632/oncotarget.1761)
Supplement: Supplementary file 2 [file oncotarget-05-1761-s002.pdf]

Spores of *Clostridium* engineered for clinical efficacy and safety cause regression and cure of tumors *in vivo*- Heap et al

Enzyme screening

Genes encoding homologs of *E. coli* NfnB and *Bacillus amyloliquefaciens* YwrO from several bacteria were cloned into an *E. coli* expression vector for screening (see Methods below). These were the NfnB homolog from *Neisseria meningitidis* MC58 ('NmeNTR', accession number Q9K022), the NfnB homolog from *Haemophilus somnus* 129PT ('HsoNTR', accession number ZP_00122352.1), the NfnB homolog from *Burkholderia fungorum* LB400 ('BfuNTR', accession number ZP_00029570.1), the YwrO homolog from *Vibrio cholerae* ('VchYwrO', accession number Q9KNX3), the YwrO homolog from *Rhodobacter sphaeroides* ('RspYwrO', accession number ZP_00007082.1), and two YwrO homologs from *Pseudomonas aeruginosa* ('PaeYwrO1', accession number Q9HUJ0; and 'PaeYwrO2', accession number Q9I4B3).

In a initial screen for functional expression of each of the cloned genes, specific menadione reductase activity was determined in lysates of *E. coli* cells containing the NfnB or YwrO homolog expression plasmid. Enzymes from these families are expected to be NAD(P)H-dependent quinone reductases regardless of their nitroreductase activity against CB1954, and a photospectrometric menadione reductase assay is available (see Methods below), so this approach provides a convenient means to assess functional expression. The specific menadione reductase activity of lysates containing NmeNTR, HsoNTR, VchYwrO, PaeYwrO1 or PaeYwrO2 were significantly elevated above the control, and hence scored as functionally expressed; while RspYwrO and BfuNTR were not.

To screen for CB1954 nitroreductase activity of each of the functionally expressed genes, lysates of *E. coli* cells containing the NfnB or YwrO homolog expression plasmid were added to reaction mixtures containing CB1954 and the concentration of CB1954 was monitored by HPLC (see Methods below). Lysates containing NmeNTR or HsoNTR caused a decrease in CB1954 concentration, indicating nitroreductase activity against CB1954. Such activity was not observed in the lysates containing VchYwrO, PaeYwrO1 or PaeYwrO2.

Attempted marker removal using FLP

In our first approach to construction of therapeutic strains, NmeNTR and *E. coli* NfnB expression cassettes were inserted into the *pyrF* locus of the *C. sporogenes* chromosome using the *pyrF*-specific Group II intron plasmid pMTL007C-E2::Csp-pyrF-595s [1, 2] as described in Methods below. Insertions made in this way also contain the marker gene *ermB*, which confers resistance to the antibiotic erythromycin, an undesirable feature in a therapeutic strain. The *ermB* gene is flanked by flippase recognition target (FRT) sites, potentially allowing marker removal using FLP recombinase [3].

The FLP recombinase expression plasmid pMTL8515-PPS-flp3 [2] was introduced into the recombinant *C. sporogenes* strain containing the integrated NmeNTR expression cassette and *ermB* by conjugation from an *E. coli* donor. Three isolated transconjugant clones containing the FLP plasmid were each cultivated overnight at 30 °C in non-selective TYG (a complex growth medium), then single colonies were obtained by plating dilutions onto non-selective TYG plates. The overnight cultures were also sub-cultured by 1:100 dilution into fresh non-selective TYG, incubated overnight at 30 °C, and single colonies were obtained by plating these second overnight cultures onto non-selective TYG plates. For each of the three isolated transconjugant clones, 16 single colonies from the first overnight culture and 34

single colonies from the second overnight culture were screened for FLP recombinase-mediated excision of the *ermB* marker by replica-plating colonies onto TYG plates supplemented with thiamphenicol and TYG plates supplemented with erythromycin. Resistance to thiamphenicol is encoded by the FLP plasmid, and resistance to erythromycin is encoded by the *ermB* gene. All clones grew on erythromycin, indicating that FLP recombinase-mediated excision of the *ermB* marker had not occurred in any clone.

METHODS

Construction of plasmids for expression of enzymes in *E. coli*

The genes encoding the candidate proteins VchYwrO, RspYwrO, NmeNTR, HsoNTR, BfuNTR, PaeYwrO1 and PaeYwrO2 were PCR-amplified from genomic DNA template or crude cell lysates using the forward and reverse primers of the same names with the suffix -F or -R respectively (Table S1). PCR products were cloned into pCR2.1 using TOPO TA Cloning Kit (Invitrogen) in accordance with the manufacturer's instructions. *E. coli* strain TOP10 (Invitrogen) was used as the host for all cloning and expression. The gene encoding HsoNTR contained an internal NdeI site, and this was silenced by overlap extension PCR in order that the NdeI site introduced by the forward primer HsoNTR-F would be unique, facilitating subsequent sub-cloning: One portion of the gene was PCR-amplified from pCR2.1::HsoNTR plasmid DNA template using the primers HsoNTR-F and HsoNTR-NdeI-A, and the remainder was PCR-amplified using the primers HsoNTR-NdeI-B and HsoNTR-R. The two PCR products were gel-purified and used as templates in an overlap extension PCR using the outer primers HsoNTR-F and HsoNTR-R. The resulting gene encodes an identical protein, but lacks the internal NdeI site.

The genes encoding NmeNTR and HsoNTR were later PCR-amplified from the relevant pCR2.1 derivatives using primers NmeNTR-F and NmeNTR-H6-R, or HsoNTR-F and HsoNTR-H6-R, respectively, yielding genes encoding proteins with carboxy-terminal six-histidine affinity tags.

Each gene was sub-cloned from pCR2.1 into the *E. coli* constitutive expression plasmid pMTL1015 [4] using restriction endonuclease sites NdeI and PstI, or NdeI and XbaI, which had been introduced into the PCR products by the primers. This placed expression of the genes under the control of the plasmid-borne promoter and RBS originating from the *mdh* gene of *E. coli*.

Enzyme characterization

Lysates of *E. coli* clones containing pMTL1015-based enzyme expression plasmids were prepared for preliminary characterization of enzyme activities. Clones were grown overnight in LB supplemented with 12.5 µg/ml tetracycline (to select for the plasmid) at 37 °C and 200 rpm shaking, and cells were harvested by centrifugation. Cell pellets were lysed either by sonication following re-suspension in 10 mM Tris HCl (pH 7.5) or using BugBuster protein extraction reagent (Novagen) in accordance with the manufacturer's instructions. For determination of CB1954 nitroreductase kinetic parameters, six-histidine affinity-tagged enzymes were purified from cell lysates purified using a Novagen His-Bind kit in accordance with the manufacturer's instructions.

Menadione reductase activity was determined by the method of Knox [5]. Cuvettes containing assay buffer (10 mM Tris HCl, pH 7.5) were pre-warmed to 37 °C. To these 10 µM menadione, 1 mM NADH or NADPH, and 70 µM bovine cytochrome C were added. Reactions were started by the addition of enzyme (either crude lysate or purified enzyme) and

prompt mixing. Reduction of cytochrome C was recorded by measuring increase in absorbance at 550 nm. Specific activities were calculated using the total protein concentrations of cell lysates, which were determined using the Bradford reagent (Sigma-Aldrich). Aliquots of cell lysates were mixed with 250 µl of Bradford reagent and incubated in the dark at room temperature for 5 min before absorbance was measured at 595 nm. Absorbance values were compared to those obtained using known concentrations of bovine serum albumin.

CB1954 nitroreductase activity was determined essentially as described by Anlezark [6]. Reaction mixtures containing 500 µM NADH or NADPH and a small quantity of enzyme (either crude lysate or purified enzyme) were assembled in pre-warmed (to 37 °C) 100 mM sodium phosphate buffer (pH 7.0), and started with the addition of 100 µM CB1954 and prompt mixing.

To determine initial reaction rates during preliminary screening of CB1954 nitroreductase candidates, aliquots of the reaction mixtures taken at various times were injected into a Partisphere SCX (100 x 4.7 mm) HPLC column and eluted isocratically at 2 ml/min with 130 mM NaH_2PO_4 (pH 5.0). Eluates were continuously monitored at 260 nm and 325 nm, and the absorbance spectra of eluting components recorded using a photodiode array detector. Peaks were identified with reference to standards and/or by their known absorbance spectra [7] and anticipated column retention times as appropriate. Rates were determined based on the decrease in the area under the CB1954 peak over time, normalised to the known starting concentration of 100 µM using the peak from the zero timepoint sample. To determine initial reaction rates and CB1954 nitroreduction products in later experiments with purified proteins, peaks were identified as before, and rates were determined based on the increase in the area under the 4-hydroxylamine derivative peak over time, normalised using enzymatically-synthesised 4-hydroxylamine standards.

Construction of recombinant *C. sporogenes*

C. sporogenes was grown in static culture at 37 °C under an anaerobic atmosphere of N_2:H_2:CO_2 (80:10:10, vol:vol:vol) in an anaerobic workstation (Don Whitley, UK) using media pre-reduced overnight under the same conditions.

NmeNTR and *E. coli* NfnB expression cassettes were initially inserted into the *C. sporogenes* chromosome using the ClosTron method as described previously [1, 2]. This approach makes directed chromosomal insertions by employing a modified bacterial Group II intron, which is a type of mobile retro-element [8]. The target site specificity of a particular Group II intron depends upon a small region of the intron RNA, and the corresponding intron-encoding DNA sequence can be rationally modified to re-target the intron [2, 9]. We previously constructed an intron variant, Csp-pyrF-595s, [1] which targets *pyrF*, a gene involved in pyrimidine biosynthesis. We chose to use this target site again in the present study, because cells in which *pyrF* is inactivated are uracil auxotrophs and effectively disabled from growth in the environment, which is an advantageous property for a potential clinical strain as discussed in the main text. The Csp-pyrF-595s intron region conferring target specificity was sub-cloned into pMTL007C-E2 [2] to construct pMTL007C-E2::Csp-pyrF-595s, a vector suitable for the Group II intron-mediated delivery of expression cassettes to the *pyrF* locus of *C. sporogenes*.

An NmeNTR expression cassette was inserted at the unique SalI site of pMTL007C-E2::Csp-pyrF-595s, so that it would be integrated into the chromosome along with the intron during retrotransposition [2]. The expression cassette comprised a synthetic NmeNTR coding sequence with codon usage optimised to suit *C. sporogenes* codon preferences fused to a promoter and RBS derived from the ferredoxin gene *fdx* of *C. sporogenes*.

Next, the plasmid was transferred into *C. sporogenes* by conjugation from a *E. coli* donor strain CA434, and transconjugant clones were selected on plates supplemented with thiamphenicol, resistance to which is encoded by the plasmid [2]. The Group II intron, including the expression cassette and an *ermB* erythromycin resistance marker, is constitutively expressed and irreversibly inserted into a specific target site in a process termed retro-transposition. Cells containing a chromosomal insertion are selected by sub-culturing transconjugant clones onto plates supplemented with erythromycin.

Our attempts to remove the *ermB* erythromycin resistance marker from the above strains using FLP recombinase (see above, Attempted marker removal using FLP) were not successful, so we circumvented the need for marker removal by using a different integration approach. Similar expression cassettes for NmeNTR and *E. coli* NfnB were each assembled in the integration vector pMTL-JH27, and then integrated into the *pyrE* locus using the procedure described previously [10]. Coding sequences for NmeNTR or *E. coli* NfnB were inserted into the multiple cloning site of pMTL-JH27 along with a promoter and RBS derived from the *fdx* gene of *C. sporogenes* via pMTL83353 [11].

Plasmids were transferred into *C. sporogenes* by conjugation from the *E. coli* donor strain CA434, and transconjugant clones were selected on plates supplemented with thiamphenicol, resistance to which is encoded by the plasmid. First, single-crossover clones were enriched by sub-culturing on thiamphenicol plates, then stable double-crossover clones were isolated by transferring cells onto plates supplemented with 5-fluoroorotic acid [10]. Loss of the plasmid (which encodes thiamphenicol resistance) was confirmed by replica-plating colonies onto unsupplemented TYG plates and TYG plates supplemented with thiamphenicol.

Characterization of recombinant *C. sporogenes*

Successful integration of the NmeNTR and *E. coli* NfnB expression cassettes in *C. sporogenes* strains N1 and E1, respectively, was verified by PCR (Fig. 2 *B*). Strains N1, E1 and the wild-type were grown overnight in TYG broth, cells were harvested by centrifugation, and genomic DNA was extracted using the Qiagen DNeasy Blood & Tissue Kit in accordance with the manufacturer's instructions, including the recommended modifications for gram-positive bacteria. PCR was performed using Phusion polymerase (NEB) and chromosome-specific primer Csp-pyrD-sF2 in combination with insert-specific primer M13F (for primer sequences, see Table S1).

C. sporogenes strains were tested for uracil auxotrophy / prototrophy by picking fresh colonies grown on TYG plates and sub-culturing them onto plates of *Clostridium botulinum* defined minimal medium MI [12] supplemented with 0 or 40 µg/ml uracil, then incubating plates at 37 °C for 24 h. The wild-type grew equally well with or without the uracil supplement, whereas the uracil auxotroph strains grew similarly to the wild-type on the uracil-supplemented plates, but showed no growth on the unsupplemented plates.
Specific menadione reductase activities of lysates of *C. sporogenes* were determined as described for *E. coli* above, except lysates were prepared from cultures in the late exponential phase of growth, rather than from overnight cultures, to avoid issues associated with sporulation and heterogeneity in overnight cultures of *C. sporogenes*. Cells were lysed using BugBuster Protein Extraction Reagent (Novagen) in accordance with the manufacturer's instructions, including rLysozyme and Benzonaze nuclease.

To evaluate the ability of strains to form spores, TYG broth was inoculated to an OD_{600} of 0.05 using a starter culture grown overnight in the same medium. After incubation at 37 °C for five days, the titre of heat-resistant colony-forming units (a measure of spore titre) was

determined by heat-treating samples of cultures at 80 °C for 20 minutes to inactivate vegetative cells, then serially-diluting and plating the samples onto TYG agar plates.

Mouse xenograft tumor model

Human colorectal HCT116 carcinoma cells were grown in DMEM medium supplemented with 10 % FBS, trypsinized, dissolved in matrigel and 50 µl injected subcutaneously at a concentration of 5.10^6 cells in the abdominal flank of adult NMRI nu/nu mice. As soon as tumors were palpable, tumor volumes were determined three times per week by measuring three orthogonal diameters (A, B and C) using calipers. Volumes (V) were calculated according to the formula V=AxBxC/6. All experiments were conducted in accordance with local institutional guidelines of the University of Leuven, approved by the animal ethics committee and procedures were according to the guidelines defined by the UKCCCR [13]. Treatment was initiated when tumors reached a volume of ~250 mm^3. Five days post spore injection (5.10^7 cfu/100 µl), sham or prodrug treatment was started. CB1954 was prepared as described previously [14] and administered intraperitoneally (i.p.) at a concentration of 15 mg/kg for five consecutive days. At the end of the follow-up period, tumors and normal tissues were excised, ground and serially diluted to determine colonization levels [15]. Tumor growth delay was determined for each individual tumor within the different groups as the time necessary to reach three times their initial volume at the start of the treatment. One-way ANOVA with a Tukey's posttest was used to determine statistical significance between the different growth curves.

REFERENCES

1. Heap JT, Pennington OJ, Cartman ST, Carter GP and Minton NP. The ClosTron: a universal gene knock-out system for the genus Clostridium. J Microbiol Methods. 2007; 70:452–464.
2. Heap JT, Kuehne SA, Ehsaan M, Cartman ST, Cooksley CM, Scott JC and Minton NP. The ClosTron: Mutagenesis in Clostridium refined and streamlined. J Microbiol Methods. 2010; 80:49–55.
3. Steiner E, Dago AE, Young DI, Heap JT, Minton NP, Hoch JA and Young M. Multiple orphan histidine kinases interact directly with Spo0A to control the initiation of endospore formation in Clostridium acetobutylicum. Mol Microbiol. 2011; 80:641-54.
4. AbuKhader MM, Heap J, De Matteis CI, Doughty SW, Minton N and Paoli M. Crystallization and preliminary X-ray characterization of the Bacillus amyloliquefaciens YwrO enzyme. Acta Crystallogr Sect F Struct Biol Cryst Commun. 2007; 63:746–750.
5. Knox RJ, Boland MP, Friedlos F, Coles B, Southan C, Roberts JJ. The nitroreductase enzyme in Walker cells that activates 5-(aziridin-1-yl)-2,4-dinitrobenzamide (CB 1954) to 5-(aziridin-1-yl)-4-hydroxylamino-2-nitrobenzamide is a form of NAD(P)H dehydrogenase (quinone) (EC 1.6.99.2). Biochem Pharmacol. 1988; 37:4671–4677.
6. Anlezark GM, Melton RG, Sherwood RF, Coles B, Friedlos F and Knox RJ. The bioactivation of 5-(aziridin-1-yl)-2,4-dinitrobenzamide (CB1954)–I. oPurification and properties of a nitroreductase enzyme from Escherichia coli–a potential enzyme for antibody-directed enzyme prodrug therapy (ADEPT). Biochem Pharmacol. 1992; 44:2289–2295.
7. Knox RJ and Chen S. Quinone reductase-mediated nitro-reduction: clinical applications. Methods Enzymol. 2004; 382:194–221.

8. Lambowitz AM and Zimmerly S. Mobile group II introns. Annu Rev Genet. 2004; 38:1–35.
9. Perutka J, Wang W, Goerlitz D and Lambowitz AM (2004) Use of computer-designed group II introns to disrupt Escherichia coli DExH/D-box protein and DNA helicase genes. J Mol Biol. 2004; 336:421–439.
10. Heap JT, Ehsaan M, Cooksley CM, Ng YK, Cartman ST, Winzer K and Minton NP. Integration of DNA into bacterial chromosomes from plasmids without a counter-selection marker. Nucleic Acids Res. 2012; 40:59.
11. Heap JT, Pennington OJ, Cartman ST and Minton NP. A modular system for Clostridium shuttle plasmids. J Microbiol Methods. 2009; 78:79–85.
12. Whitmer ME, Johnson EA. (1988) Development of improved defined media for Clostridium botulinum serotypes A, B, and E. Appl Environ Microbiol. 1988; 54:753–759.
13. Workman P, Balmain A, Hickman JA, McNally NJ, Rohas AM, Mitchison NA, Pierrepoint CG, Raymond R, Rowlatt C, Stephens TC and Wallace J. UKCCCR guidelines for the welfare of animals in experimental neoplasia. Lab Anim. 1988; 22:195–201.
14. Djeha AH, Hulme A, Dexter MT, Mountain A, Young LS, Searle PF, Kerr DJ and Wrighton CJ. Expression of Escherichia coli B nitroreductase in established human tumor xenografts in mice results in potent antitumoral and bystander effects upon systemic administration of the prodrug CB1954. Cancer Gene Ther. 2000; 7:721–731.
15. Lambin P, Theys J, Landuyt W, Rijken P, van der Kogel A, van der Schueren E, Hodgkiss R, Fowler J, Nuyts S, de Bruijn E, Van Mellaert L and Anné J. Colonisation of Clostridium in the body is restricted to hypoxic and necrotic areas of tumours. Anaerobe. 1998; 4:183–188.
