# Supplementary material for: Spores of Clostridium engineered for clinical efficacy and safety cause regression and cure of tumors in vivo
Source: Oncotarget. 2014 Jan 12;5(7):1761–9. doi: 10.18632/oncotarget.1761 (PMC4039107; doi:10.18632/oncotarget.1761)
Supplement: Supplementary file 3 [file oncotarget-05-1761-s003.pdf]

Spores of *Clostridium* engineered for clinical efficacy and safety cause regression and cure of tumors *in vivo*- Heap et al

Table S1: List of oligonucleotide primers.

Primer	Sequence (5′-3′)
VchYwrO-F	CAGAAGGATTCAGCATGTGCGCAACTTGGTCAACCGAGAGCAAACGAAA
VchYwrO-R	AGTCTAGAAGAGACGACACTGCTACTGAGAAAATCACTGGTCA
RspYwrO-F	CAGAAGGATTCAGCATGTGGAATTCCCCTTTTCGCGGCTTGTCG
RspYwrO-R	AGTCTAGAAGAGGCCTGCACGAGCGGGACGG
NmeNTR-F	ATGACAGTATTAAGCAAAGAGCAGGTTCTATCC
NmeNTR-R	TTATGCCCAAATAACGGTTTCTTCCAA
HsoNTR-F	ATGACGACTATTTCAAAAGAACACGTGCTGGATA
HsoNTR-R	TTATTTAACCCAAGTGACGATTTCATCAAGG
BfuNTR-F	CAGAAGGATTCAGCATATGCTGCCGGGCCGGCTGACATT
BfuNTR-R	AGCTGCAGAGCGTTGAATACTCTGCGGCACGCTGGTCC
PaeYwrO1-F	CAGAAGGATTCAGCATATGAACGTACTGATCG
PaeYwrO1-R	AGCTGCAGAGGGGGGCGTCTTGGCTTGACTGGC
PaeYwrO2-F	CAGAAGGATTCAGCATATGCATGCCCTGATCGTCGTCGCTC
PaeYwrO2-R	AGTCTAGAAGCGGGGGTCAGGCCTCCAGCGG
HsoNTR-NdeI-A	GCGTTTTCATGTGTTCTGCT
HsoNTR-NdeI-B	AGCAGAACACATGAAAACGC
NmeNTR-H6-R	AGCTGCAGAGTTAATGATGATGATGATGATGTGCCCAAATAACGGTTTCTTCCAA
HsoNTR-H6-R	AGCTGCAGAGTTAATGATGATGATGATGATGTTTAACCCAAGTGACGATTTCATCAAGG
M13F	TGTAAAACGACGGCCAGT
Csp-pyrD-sF2	GAAGACTTAGAAAATTATATGAAAGAAGAAGGT
